# Analysis of sublethal arsenic toxicity to *Ceratophyllum demersum*: subcellular distribution of arsenic and inhibition of chlorophyll biosynthesis

**DOI:** 10.1093/jxb/erw238

**Published:** 2016-06-23

**Authors:** Seema Mishra, Matthias Alfeld, Roman Sobotka, Elisa Andresen, Gerald Falkenberg, Hendrik Küpper

**Affiliations:** ^1^CSIR-National Botanical Research Institute, Plant Ecology & Environmental Science Division, Rana Pratap Marg, Lucknow 226 001 (U.P.), India; ^2^Universität Konstanz, Mathematisch-Naturwissenschaftliche Sektion, Fachbereich Biologie, Postfach M665, D-78457 Konstanz, Germany; ^3^Deutsches Elektronen-Synchrotron (DESY), Photon Science, Notkestr. 85, 22603 Hamburg, Germany; ^4^Centre Algatech, Institute of Microbiology, Academy of Sciences of the Czech Republic, CZ-379 81 Třeboň, Czech Republic; ^5^Institute of Plant Molecular Biology, Department of Biophysics and Biochemistry of Plants, Biology Centre of the AS CR, Branišovská 31/1160, CZ-370 05 České Budějovice, Czech Republic; ^6^University of South Bohemia, Faculty of Science, Branišovská 31, CZ-370 05 České Budejovice, Czech Republic

**Keywords:** Arsenic, arsenic toxicity, chlorophyll biosynthesis, chlorophyll precursors, subcellular distribution of arsenic, synchrotron micro-X-ray fluorescence

## Abstract

Arsenic (As) pollution is a serious concern worldwide. Recent studies under environmentally relevant conditions revealed that, in the aquatic plant *Ceratophyllum demersum*, pigments are the first observable target of toxicity, prior to any effect on photosynthetic parameters or to oxidative stress. Lethal toxicity was initiated by a change of As species and their distribution pattern in various tissues. Here, the localization of As was investigated at the subcellular level through X-ray fluorescence using a submicron beam and a Maia detector. Further, it was possible to obtain useful tissue structural information from the ratio of the tomogram of photon flux behind the sample to the tomogram of Compton scattering. The micro-X-ray fluorescence tomograms showed that As predominantly accumulated in the nucleus of the epidermal cells in young mature leaves exposed to sublethal 1 µM As. This suggests that As may exert toxic effects in the nucleus, for example, by interfering with nucleic acid synthesis by replacing phosphorous with As. At higher cellular concentrations, As was mainly stored in the vacuole, particularly in mature leaves. An analysis of precursors of chlorophyll and degradation metabolites revealed that the observed decrease in chlorophyll concentration was associated with hindered biosynthesis, and was not due to degradation. Coproporphyrinogen III could not be detected after exposure to only 0.5 µM As. Levels of subsequent precursors, for example, protoporphyrin IX, Mg-protoporphyrin, Mg-protoporphyrin methyl ester, and divinyl protochlorophyllide, were significantly decreased at this concentration as well, indicating that the pathway was blocked upstream of tetrapyrrole synthesis.

## Highlight

At sublethal toxic concentrations, arsenic is predominantly localized in the nucleus but is already able to inhibit chlorophyll biosynthesis upstream of coproporphyrinogen III.

## Introduction

Though arsenic (As) is ubiquitous in the environment, its elevated concentrations in water and soil in many areas of the world are of serious environmental and human health concern. Both natural and anthropogenic activities have contributed to As contamination (Smedley and Kinniburgh, 2002; Mondal *et al.*, 2006). The groundwater of many countries, particularly in the Indian subcontinent, are naturally highly enriched with As and pose a toxicity risk through contamination of drinking water and the food chain. Agricultural fields are the net sink for the thousands of tons of As transferred each year through contaminated irrigation water (Ali, 2003; Neumann *et al.*, 2011). The typical As concentration in the soil solution of paddy fields varies from 0.01 to 3 µM. However, As concentrations as high as 33 µM have been reported in a paddy field irrigated with As-laden groundwater (Panullah *et al.*, 2009; Zhao *et al.*, 2009). Panullah *et al.* (2009) reported severe yield loss in crops grown in As-contaminated fields. Therefore, both food quality and food security are at risk in As-contaminated areas. Understanding the mechanism of As toxicity is crucial for finding a sustainable solution to the problem, and determining the *in planta* distribution and speciation of As are important steps in this process.

Inorganic arsenate [HAsO_4_^2−^ or As(V)] and arsenite [H_2_AsO_3_^−^ or As(III)] are the common forms present in aquatic and terrestrial environments. Arsenate, the predominant form in aerobic environments, is taken up by the plants through phosphate transporters (Asher and Reay, 1979). Arsenite, in contrast, predominates in anaerobic environments and is absorbed through nodulin 26-like intrinsic aquaporins (Isayenkov and Maathuis, 2008; Ma *et al.*, 2008). Whether exposed to As(V) or As(III), it is As(III) that is the predominant form inside the plant, suggesting efficient *in planta* reduction of As(V) (Pickering *et al.*, 2000, 2006; Xu *et al.*, 2007; Mishra *et al.*, 2013). A homologue of yeast arsenate reductase ACR2 has been identified in plants (Zhao *et al.*, 2010). However, recent studies showed that *ACR2*-like genes have no function in As metabolism in plants (Liu *et al.*, 2012; Chao *et al.*, 2014). Instead, another novel arsenate reductase, termed HAC1 (Chao *et al.*, 2014) or ATQ1 (Sánchez-Bermejo *et al.*, 2014), has been shown to play a crucial role in As metabolism in *Arabidopsis thaliana* through As(III) efflux from the roots, thus minimizing As in shoots (Chao *et al.*, 2014). Besides efflux, As(III) has a high affinity for thiol groups, and most As in root tissues is complexed with glutathione and/or phytochelatins (PCs) and stored within the vacuole (Zhao *et al.*, 2009; Song *et al.*, 2010; Moore *et al.*, 2011). Yet some As becomes loaded into the xylem and contributes to significant accumulation in shoots (including seeds) and causes severe toxicity (Carbonell-Barrachina *et al.*, 1997; Shri *et al.*, 2009; Zhao *et al.*, 2009). In earlier studies, As-induced production of reactive oxygen species (ROS) leading to oxidative stress has been suggested as the main mechanism of As toxicity (Hartley-Whitaker *et al.*, 2001; Requejo and Tena, 2005). However, our recent study at physiologically relevant concentrations of As showed that oxidative stress was not the reason for reduced growth or inhibition in photosynthesis (Mishra *et al.*, 2014). A decline in photosynthetic pigments was the earliest event leading to growth inhibition, and was evident at the lowest As exposure concentration (0.5 µM As(V)) tested in the study. Inhibition of photosynthetic electron transport and the photosystem II reaction centre occurred later and at higher As concentrations. Oxidative stress was the final event, and a result of malfunctioning photosynthesis (Mishra *et al.*, 2014). This switchover from sublethal to lethal toxicity correlated with a change in As species and in the pattern of As distribution, that is, an increase in the concentration of As, mainly as As(III), in mesophyll cells (Mishra *et al.*, 2013). The decrease in photosynthetic pigments and retarded plant growth at a much lower concentration indicate that As is much more toxic to plants than previously supposed, and is due to a different mechanism.

In the present study, we analysed the localization of As in the leaves of *C. demersum* at the subcellular level to better understand the intracellular mechanism of As toxicity. The localization of As was analysed through synchrotron X-ray fluorescence (XRF) tomography using a submicron beam and the Maia detector, a new-generation XRF detector featuring a high maximum count-rate capacity (up to 10 million counts per second). This high capacity, in combination with its efficient control software and direct connection between the detector control unit and the scanning motor encoders, allows for much faster data acquisition than traditional systems (Ryan *et al.*, 2010). We further analysed the precursors of chlorophyll at different As concentrations with the aim of finding the target of inhibition in the pigment biosynthesis pathway.

## Methods

### Plant material and cultivation

*C. demersum* plants were grown in nutrient solution as described earlier (Mishra *et al.*, 2013). Briefly, the nutrient solution was aerated continuously and constantly exchanged, with a 2L resident volume. The temperature and light conditions were a 14h day length, 24°C day/20°C night temperatures, and a sinusoidal light cycle with maximum irradiance of approximately 100 µmol·m^−2^·s^−1^ supplied by ‘daylight’ fluorescent tubes (Dulux L 55W/12–950; Osram). For the experiments, plants of similar weight (approximately 1g) were chosen and exposed to As(V) (0–20 µM) prepared in nutrient solution using the salt Na_2_HAsO_4_ (Alfa Aesar).

### Localization of As through synchrotron µXRF

The leaves of plants exposed to 1 or 5 µM As(V), representing sublethal and lethal concentrations, respectively (Mishra *et al.*, 2013), were used for synchrotron µXRF mapping of As. The plants were washed thoroughly with nutrient solution without micronutrients and As. One fully developed leaf from the fourth to the sixth whorl and one from the 15th whorl (counted from the apex) were sampled and termed as young-mature leaf and mature leaf, respectively. For sample preparation, ultraclean glass capillaries (1mm diameter, 0.1mm wall thickness; Hilgenberg) were cut to adequate size and filled with water. The leaf was carefully inserted into the water-filled capillary, avoiding any damage to the leaf, and the filled capillary was subsequently fixed on a bespoke sample holder. The capillaries containing leaves were immediately shock-frozen in supercooled isopentane (−140°C) and stored in liquid nitrogen until the analysis.

### Instrument setup for synchrotron µXRF

The µXRF measurements were done at beamline P06 of the synchrotron PETRA III at the Deutsches Elektronen-Synchrotron (DESY) in Hamburg. The X-ray beam was generated in a 2 m Spectroscopy undulator U32 and monochromatized using a cryogenically cooled Si(111) Double Crystal Monochromator at 15 keV with approximately 1.4×10^−4^ dE/E band pass. Focusing was achieved using Kirkpatrick-Baez (KB) mirrors to an ~400×600nm spot size. A 384-element Maia detector, placed in a backscatter geometry, was used to collect the emitted XRF photons from the sample. The intensity of the primary beam was monitored with an ionization chamber before the KB system, and the intensity of the transmitted radiation was monitored with a Passivated Implanted Planar Silicon (PIPS) diode behind the sample. The Maia detector enables ultrafast data acquisition with dwell times below 1ms per pixel. The sample was cooled by a cryostream (Oxford Cryosystems) from the top to about 100K. µXRF tomography was done approximately 3.5mm above the branching point of the leaf with a step size of 0.25 µm and a typical dwell time of 1–3ms per step. More than 3600 line scans were measured, with the sample being rotated in steps of 0.1°, yielding a 360° tomogram. The resulting tomograms, featuring the leaf and the entire capillary, were reconstructed using a variation of the maximum likelihood expectation maximization (MLEM) algorithm implemented in XRDUA (De Nolf *et al.*, 2014). Finally, a multi-element standard (containing 1mM each of Na_2_HAsO_4_, CdCl_2_, CrCl_3_, CuCl_2_, NaFe(III)-EDTA, NiCl_2_, and ZnCl_2_ in 20% glycerol + 5% HCl to final concentration), prepared in the same type of capillary and shock-frozen like the plant samples, was measured in the identical geometry. It was used to relate XRF intensities to micromolar concentrations, and to correct for matrix effects as described previously (Mishra *et al.*, 2013).

### Reconstruction of the tissue cross section

The virtual cross section of the samples showing tissue structures was created in ImageJ using tomograms of the flux behind the sample, that is, diminished by absorption (in the following called ‘absorption’) and Compton scattering of respective As µXRF measurements at 15 keV. A ratio of absorption to Compton scattering was calculated after enhancing the contrast of each image, and the contrast of the obtained ratio image was further enhanced using a contrast limited adaptive histogram equalization (CLAHE) algorithm.

### Analysis of chlorophyll precursors through HPLC

About 100mg of control and As-exposed (up to 20 µM) frozen plant material was ground in liquid nitrogen to fine powder and extracted in 200 µl of 75% MeOH. The extraction was repeated three times and all supernatants were pooled. The 150 µl of extract was injected into an HPLC system (Agilent 1200) and chlorophyll precursors were separated and detected by two connected fluorescence detectors (FLD1 and FLD2). The first fluorescence detector was set to 400/620nm (excitation/emission wavelengths) for 0–11min, 440/640nm for 11–20min, and 400/630nm for 20–30min. The second fluorescence detector was set to 416/595nm throughout the experiment (Pilný *et al.*, 2015).

## Results and discussion

### Technical advances

Using a combination of submicron beam size (400×600nm) and Maia, an efficient XRF-detector (Ryan *et al.*, 2010), it was possible to distinguish the cells in leaf tissue much more clearly than in our previous work (Mishra *et al.*, 2013), and enabled us to resolve the subcellular distribution of As accumulation. The Maia detector allows for faster scanning than traditional systems and permits the *in situ* examination of elements in fresh and hydrated plant tissues (Kopittke *et al.*, 2011, 2012) without any observable damage. Nevertheless, a measuring time of 10–14h (depending on the size of sample) was required for µXRF imaging of As with the high lateral and spatial resolution used in the present study, which may damage hydrated plant tissues. To avoid at least any thermal damage from the beam, the tissues were analysed in a shock-frozen hydrated instead of a fresh (non-frozen) state. Thus, in the present study, no beam damage or movement of the sample during measurement was observed.

**Fig. 1.**
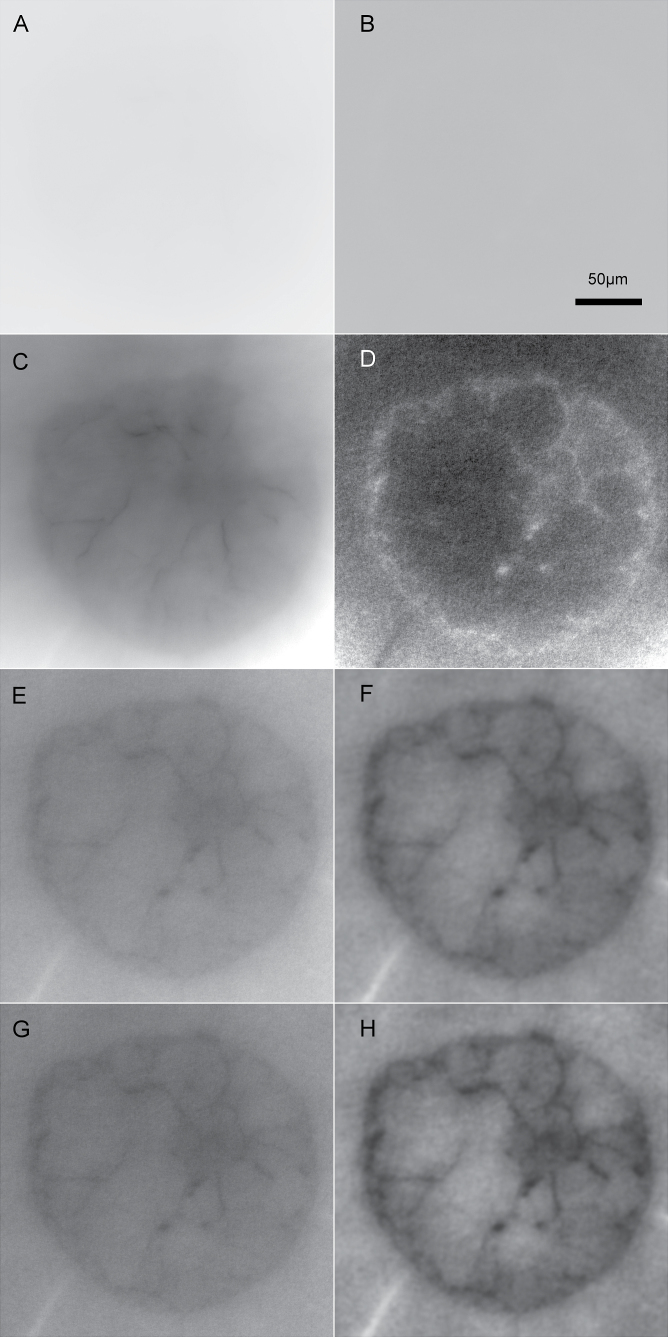
Resolving tissue structures by absorption and Compton scattering tomograms. As an example, a sample of a control leaf at 0 µM As is shown. The tomograms were provided in 32 bit mode with the shown area corresponding to 1000×1000. The scale bar in panel B applies to all images. (**A**) Tomogram of sample absorption represented by the photon flux after the sample. (**B**) Tomogram of the Compton scattering peak, recorded by the Maia detector in backscatter geometry like all element distribution maps. (**C, D**) Absorption and Compton tomograms, respectively, after general linear contrast stretching. (**E**) Tomogram of flux divided by tomogram of Compton, with linear contrast stretching then applied. (**F**) The flux/Compton ratio tomogram after application of an FFT bandpass filter with 400 pixels upper and 10 pixels lower limit, with linear contrast stretching then applied. (**G**) The flux/Compton ratio tomogram after application of the CLAHE algorithm with 1024 bins, slope 3. (**H**) The flux/Compton ratio tomogram after application of the FFT filter as in panel F, and then the CLAHE algorithm as in panel G, and finally linear contrast stretching.

A common difficulty in interpreting µXRF results in tomograms of biological tissues is the lack of directly comparable structural information. The problem of getting structural information in this type of sample originates from the composition of biological samples: almost all of their mass consists of the light elements H, C, N, and O. Therefore, looking at raw data of absorption or scattering of such samples typically does not show any structures (Fig. 1A, B). In the current work, the good statistics of the signal representing transmitted radiation (i.e. the photons not absorbed in the sample), as well as the strong scatter signal that is typical for the backscatter geometry of the Maia detector alignment, allowed us to extract structural tissue information despite these problems. As a basis, absorption and Compton scattering signals were processed like regular µXRF signals (see Methods) to generate 32 bit greyscale tomograms, which seemingly did not contain meaningful structural information (Fig. 1A, B). Contrast stretching revealed structural features in both signals, with higher scattering and lower absorption inside the cells compared to the surrounding aqueous medium, which can be explained by the lower atomic mass of carbon in organic substances compared to oxygen in water (Fig. 1C, D). Compton is more sensitive to light elements, and absorption is more sensitive to heavier elements. Forming a ratio using both tomograms combined their information into one clearer image (Fig. 1E). This could further be optimized by application of a fast Fourier transform (FFT) bandpass filter (Fig. 1F) and a CLAHE algorithm (Fig. 1G), leading to a structural tomogram that clearly showed all tissue types in the sample and thus allowed for a reliable interpretation of the element distributions (Fig. 1H). The contrast observed is based on the different ratios of the incoherent scattering cross section and the total absorption coefficient for water (H_2_O: 0.17cm^2^·g^−1^/1.67cm^2^·g^−1^ = 0.10) and plant material largely consisting of cellulose as a polymer of glucose (C_6_H_12_O_6_: 0.16cm^2^·g^−1^/1.33cm^2^·g^−1^ = 0.122) at 15 keV (Berger *et al.*, 2010).

### Intracellular arsenic distribution at sublethal and lethal toxicity

**Fig. 2.**
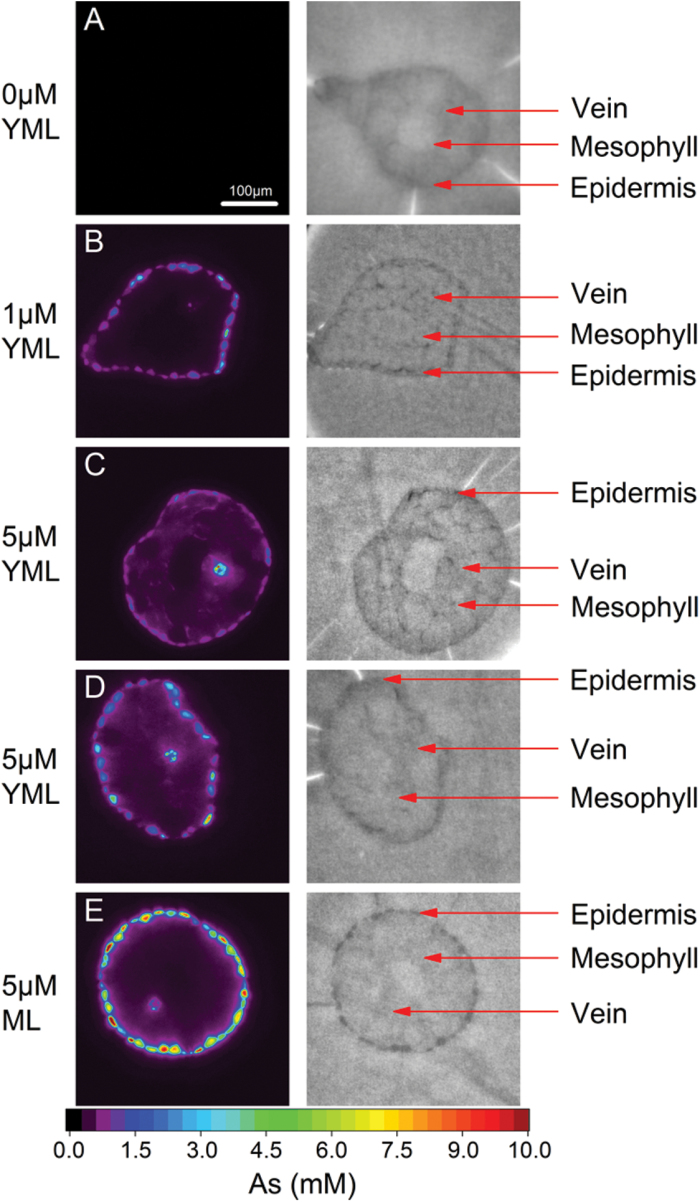
X-ray fluorescence microtomograms showing As distribution in leaves of *C. demersum* after 2 weeks of growth in control conditions or exposed to As (left panel), and the corresponding tissue structures constructed using flux and Compton tomograms (right panel). (**A**) Control young-mature leaf; (**B**) 1 µM As-exposed young-mature leaf; (**C**) 5 µM-As exposed young-mature leaf; (**D**) 5 µM-As exposed young-mature but older than above mentioned; (**E**) 5 µM-As exposed mature leaf.

The tomographic reconstruction of the µXRF measurements showed that at lower concentrations (1 µM As) most of the As was located in the epidermis of young mature leaves, very little As was in the vein, and the As level in the mesophyll was undetectable (Fig. 2B). When the concentration of As was increased (5 µM), a leaf of the same age showed significant amount of As in the mesophyll and increased levels in the vein (Fig. 2C). With an increase in tissue age (the leaf shown in Fig. 2D was older than in Fig. 2B and Fig. 2C), the level of As in the mesophyll and vein decreased, whereas it increased in the epidermis. There was no measurable amount of As in the mesophyll of the mature leaf although the epidermis contained the maximum level of As observed in the present study (Fig. 2E). Therefore, the measurements in the present study, performed at higher resolution, confirmed our previous finding that the epidermis is the main storage site of As, and the capacity of the epidermis (which is greater in mature than young-mature leaves) is crucial for increasing As concentration in other tissues as well as the onset of lethal toxicity (Mishra *et al.*, 2013, 2014).

**Fig. 3.**
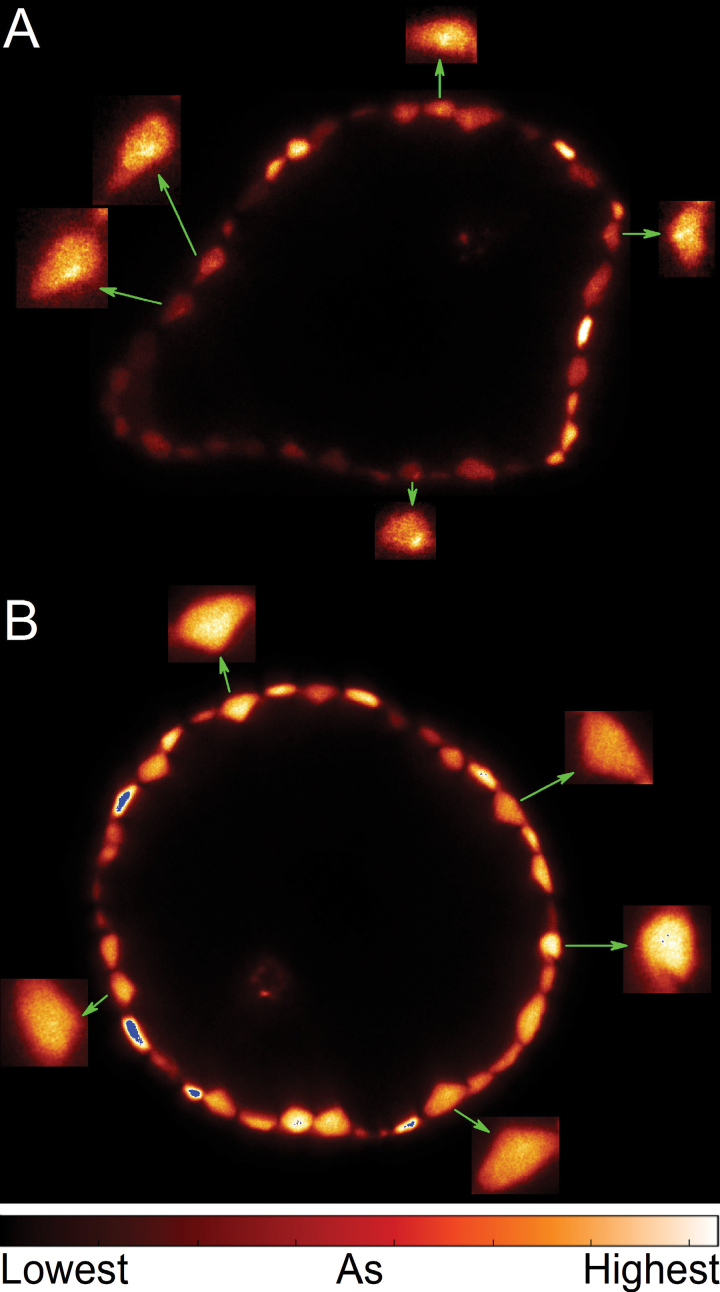
X-ray fluorescence microtomograms showing intracellular details of As distribution in leaves of *C. demersum* after 2 weeks of As treatment. (**A**) 1 µM-As exposed young mature leaf; (**B**) 5 µM As-exposed mature leaf. The arrows indicate enlarged views of selected cells to show intracellular details of As distribution.

The higher resolution measurement did not only show the individual cells much more clearly than the earlier measurements, but for the first time revealed subcellular details of As distribution in intact leaf tissues. First, it should be noted that As clearly accumulated intracellularly, and not in the cell walls. Furthermore, a differential accumulation pattern was found in epidermal cells depending on the concentration of As. At lower cellular concentration, As predominantly accumulated in a single large organelle towards the side of the cells (Fig. 3A). Given that only the nucleus has this combination of number, size, and position, it is clear that As accumulation mainly occurred in the nucleus at this concentration. In contrast, at higher cellular As concentrations, the vacuole was the main site of As accumulation (Fig. 3B). Competition with phosphate during phosphorylation reactions and replacement of P from biomolecules was suggested to be one of the mechanisms of As toxicity in the form of As(V) (Finnegan and Chen, 2012). The strong accumulation of As in the nucleus suggests *in vivo* relevance for the toxic replacement of P by As in the DNA molecule (proposed by Dixon and Webb, 1958; Westheimer, 1987; Dixon, 1997; plant-related review Patra *et al*., 2008). The result of As accumulation in the nucleus indicates that As may cause DNA damage at substantially lower, physiologically relevant concentrations, because As(V)-esters are often unstable and short lived in physiological conditions (Baer *et al.*, 1981; Finnegan and Chen, 2012). However, this is not yet proof of As binding to DNA; alternatively, As could also bind to other ligands in the nucleus and indirectly cause the known As genotoxicity. Obtaining conclusive evidence will be a task for future work, but currently no reliable method to do this exists. Measuring As in extracted DNA would not be an option because of the instability of As-DNA, and there is no method available that could prove the existence of As-DNA without extraction. Because As(V) can easily cross internal membranes through the various P transporters, resulting in rapid equilibrium of As in the entire cell, it can hamper cellular metabolism by replacing P from all possible sites. Therefore, the sugar-phosphate backbone of nucleic acids in the nucleus is an obvious target.

The accumulation of As in the nucleus observed at lower cellular As concentration, compared to the As-filled vacuole at higher As concentration, can be explained in terms of available ligands. Because of the transport of As(V) by P transporters that exist in all membranes, the final As concentration in different cellular compartments will not be determined by the transporters but by ligands of As that do not cross the membranes. In view of this principle, the results of our measurements first of all show that the nucleus contains ligands with high affinity for As that do not cross the membrane before or after binding of As. Whereas only As(V) can possibly substitute for P in DNA, only As(III) can bind to thiols and therefore be stored as As–PC complexes in vacuoles. Tissue-specific As speciation at different concentrations of As and ages of tissues showed a higher proportion of As(V) in the epidermis of young-mature leaves whereas the epidermis of mature leaves contained mostly thiol-bound As (Mishra *et al.*, 2013). Furthermore, the epidermis of mature leaves contained maximal As, observed by µXRF measurements in the same study. Probably at higher cellular concentration the nuclear As cannot be distinguished due to the high concentration of As in vacuole of mature leaves. Once the nucleus becomes saturated with As, the force driving As into that organelle will cease. The nucleus may become saturated when the ligands (proteins and nucleic acids) in the nucleus are saturated, which will lead to more rapid outflow of further As entering the nucleus. Thus, other locations become the dominant As stores, and in plant cells this seems to be the vacuole, comprising the largest volume of a plant cell. The speciation of the nuclear versus vacuolar As cannot be selectively measured with the current spatial resolution of µXAS.

In plants, As(III) is the predominant form of As due to rapid reduction of As(V) (Zhao *et al.*, 2009). *C. demersum* also accumulates 83–90% As(III) upon exposure to As(V), but a higher proportion of As(V) was observed in young-mature leaves compared to mature leaves (Mishra *et al.*, 2013). Reduction of As(V) to As(III), complexation with thiol, and subsequent sequestration of complexes in the vacuole appears to be the main detoxification mechanism in plants (Song *et al.*, 2010; Moore *et al.*, 2011; Mishra *et al.*, 2008, 2013). In absence of sufficient vacuolar sequestration, the As complexes may dissociate in the cytosol, thus increasing the concentration of As(III). The weakly bound As(III) may inhibit arsenate reductase, leading to enhanced concentration of As(V). It seems that any amount of As left unreduced and thus uncomplexed at a given time may replace P from available sites. Considering its higher *in planta* and cellular mobility, As(V) may be more toxic than As(III) at lower concentrations. Intracellular detail about the localization of As in mesophyll cells may reveal the mechanism of initiation of As toxicity at lower concentrations. However, the beam damage due to the high intensity X-ray required for achieving such sensitivity for mesophyll cells, which have lower As concentrations than the epidermis, at present prevents the success of such experiments.

### The mechanism of As-induced inhibition of chlorophyll biosynthesis

While the µXRF measurements have revealed As binding in the nucleus at surprisingly low As stress levels, our previous study revealed that pigments were the first target of As toxicity when comparing various processes (Mishra *et al.*, 2014). A decrease in chlorophylls preceded all changes in measured parameters of photosynthesis biophysics (i.e. activity of photosystem II reaction centre, linear photosynthetic e^−^ transport, and regulation of non-photochemical exciton quenching), starch accumulation, respiration, nutrient uptake, and the formation of ROS as a stress phenomenon. Moreover, this decrease in chlorophyll levels also occurred at much lower As exposure concentrations, that is, 0.5 µM, than the other processes (Mishra *et al.*, 2014). The information obtained from quantitative µXRF tomograms in the present study as well as in a previous report (Mishra *et al.*, 2013) demonstrates that the level of As in mesophyll cells would be extremely low at 0.5 µM. Thus, As affects the accumulation of chlorophyll complexes immediately after reaching the mesophyll.

**Fig. 4.**
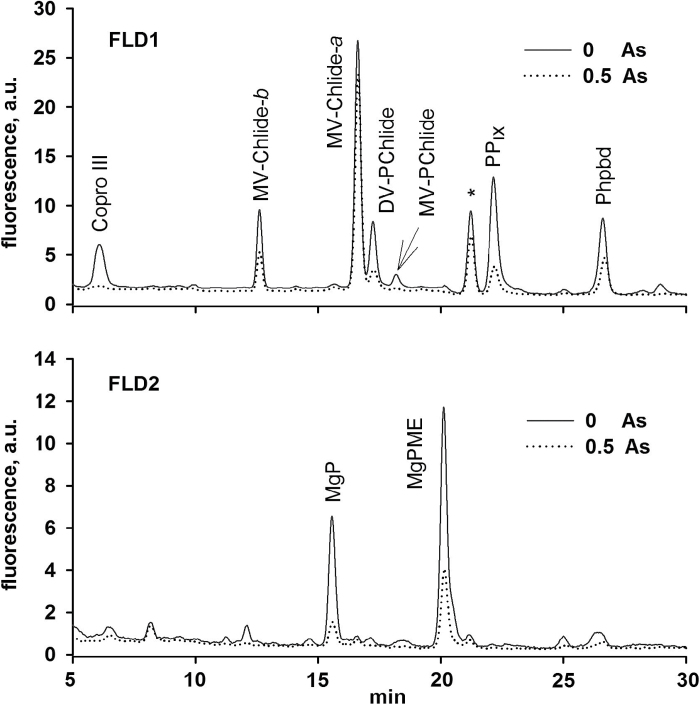
HPLC chromatograms of control and 0.5 µM arsenic-exposed plant showing inhibition of chlorophyll precursors. Extracted pigments were separated by HPLC equipped by two fluorescence detectors (FLD1 and FLD2). Each detector was set to wavelengths allowing them to detect indicated chlorophyll precursors (Pilný *et al.*, 2015). Copro III, coproporphyrin(ogen) III; PP_IX_, protoporphyrin(ogen) IX; MgP, Mg-protoporphyrin; MgPME, Mg-protoporphyrin methyl ester, MV-Chlide-*a* and *-b*, monovinyl chlorophyllide *a* and *b*; MV-PChlide, monovinyl protochlorophyllide *a*; DV-Pchlide, divinyl protochlorophyllide *a*; Phpbd, pheophorbide. The asterisk (*) indicates an unknown chlorophyll derivate.

To investigate the mechanism responsible for the rapid loss of chlorophyll, levels of precursors in chlorophyll biosynthesis were analysed in *C. demersum* plants treated with As. The method used (Pilný *et al.*, 2015) allowed us to quantify all precursors of the tetrapyrrole pathway down from coproporphyrinogen III (Copro III), an early precursor shared by both heme and chlorophyll synthesis. Copro III is converted in two steps to protoporphyrin IX, and insertion of Fe or Mg into protoporphyrin IX leads to the formation of heme or Mg-protoporphyrin, respectively. Interestingly, even at the lowest concentration of As that was tested (0.5 μM), Copro III was almost undetectable (Fig. 4). A very strong decrease was observed for levels of all other precursors (Fig. 4) except monovinyl chlorophyllide *a*. However, this last chlorophyll precursor can also originate from chlorophyll recycling and its level does not reflect the inhibition of *de novo* chlorophyll synthesis (Kopečná *et al.*, 2015).

**Fig. 5.**
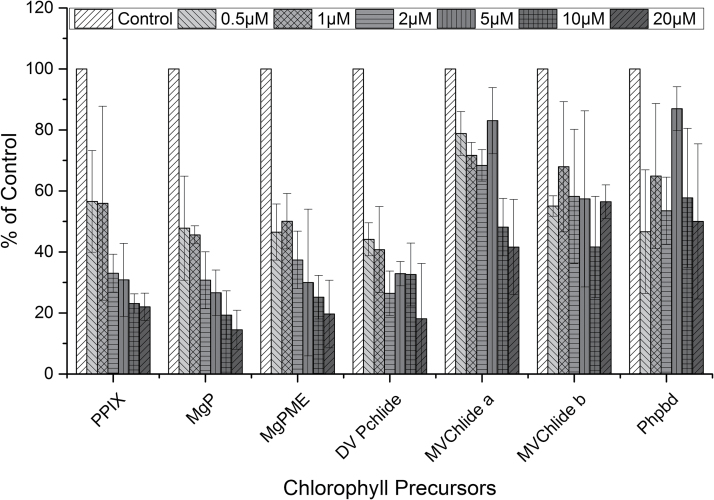
As concentration-dependent changes in chlorophyll precursors and pheophorbide (Phpbd) in *C. demersum* leaves with respect to Control leaves. Coproporphyrin(ogen) III was not detectable under any tested As concentration (see Fig. 4). PPIX, protoporphyrin(ogen) IX; MgP, Mg-protoporphyrin; MgPME, Mg-protoporphyrin methyl ester, DV Pchlide, divinyl protochlorophyllide *a*; MVChlide *a* and *b*, monovinyl chlorophyllide *a* and *b.* The values are averages ±SE of three independent experiments.

The increase in As concentration further deepened the lack of chlorophyll precursors as well as the level of pheophorbide *a* (Fig. 5), which is an intermediate of chlorophyll catabolism (review by Hortensteiner, 2006). This indicates that As does not accelerate the degradation of chlorophyll–protein complexes, and the inhibition of the tetrapyrrole biosynthetic pathway is very likely responsible for the observed chlorophyll bleaching in As-treated cells. According to the data presented here, As inhibits the pathway upstream of Copro III, which is in contrast to Co and Mn, which have previously been shown to affect later steps of chlorophyll biosynthesis in cyanobacteria (Csatorday *et al.*, 1984). The enzyme 5-aminolevulinic acid dehydratase was studied previously in plants for its sensitivity to heavy metals (Prasad and Prasad, 1987; Gupta *et al.*, 2013). It has been speculated that treatment with heavy metals like Hg or Co replaces one or more of the Mg atoms required for the formation of the highly active octameric enzyme complex (Kervinen *et al.*, 2000; Gupta *et al.*, 2013). Although the sensitivity of 5-aminolevulinic acid dehydratase to As had been observed in *Zea mays* leaves, much higher As concentrations have been reported (Jain and Gadre, 2004) than in the present study. Thus, it seems that some other enzymatic step at the beginning of the tetrapyrrole pathway is extremely sensitive to As. Because Copro III is also a precursor for heme biosynthesis, an adverse effect of As on the accumulation of cytochromes cannot be excluded. This should have affected, however, the electron flow in photosynthesis and respiration, which was not observed at the very low As concentrations that have been shown to inhibit chlorophyll biosynthesis (Mishra *et al.*, 2014).

The insight into As-induced inhibition of chlorophyll biosynthesis in *Ceratophyllum* (having no root and with chloroplasts in the epidermal layer of leaves) in the present study is particularly important to show the sensitivity of the chlorophyll biosynthesis pathway to low As concentrations. It remains to be investigated whether other species, for example, rice (*Oryza sativa*) as a typically As-affected crop, require higher As concentrations for inhibition of chlorophyll biosynthesis. At higher As concentrations, the other impacts of As, such as the replacement of P and subsequent damage to DNA or other modes of root toxicity, may overshadow the effect on chlorophyll.

## Conclusions

From the results presented here it can be concluded that As is much more toxic than previously thought at a concentration which can be easily attained in shoots of crop plants. At these low but toxic concentrations, As inside the cells mostly accumulated in the nuclei of the epidermal cells, but the small amount of As reaching chloroplasts nevertheless caused a strong and specific inhibition of tetrapyrrole biosynthesis. This will ultimately lead to the previously reported yield loss. In terms of methodology, this work has shown that tissue structural information that is useful for interpreting the location of µXRF signals can be obtained from the ratio of the absorption to the Compton scattering signal.
